# A modified endoluminal vacuum therapy system for salvage management of recurrent anastomotic leak after revisional bariatric surgery

**DOI:** 10.1093/jscr/rjag669

**Published:** 2026-08-06

**Authors:** Silvana Alexandra Valencia Valverde, Ronnal Patricio Vargas, Madeleine Elizabeth Carrion Correa, Miguel Angel Moyon, Fernando Xavier Moyon, Naysi Cristina Zambrano, Mateo Sebastian Valencia

**Affiliations:** Department of Surgery, General Hospital San Francisco, Quito 170120, Ecuador; Department of Surgery, General Hospital San Francisco, Quito 170120, Ecuador; Pontifical Catholic University of Ecuador, Quito 170525, Ecuador; Department of Surgery, General Hospital San Francisco, Quito 170120, Ecuador; Department of Surgery, General Hospital San Francisco, Quito 170120, Ecuador; Department of Surgery, General Hospital San Francisco, Quito 170120, Ecuador; Universidad UTE, Quito, Ecuador

**Keywords:** bariatric surgery, endoluminal vacuum therapy, anastomotic leak, revisional surgery, gastrointestinal fistula

## Abstract

Endoluminal vacuum therapy (EVT) has emerged as a minimally invasive and effective approach for the management of gastrointestinal anastomotic leaks, particularly in complex or refractory cases. Meta-analyses report pooled clinical success rates of ~80%–85%, with evidence suggesting higher success compared with self-expanding metal stents. We report the case of a 55-year-old female with a history of sleeve gastrectomy who underwent revisional bariatric surgery complicated by multiple anastomotic failures, open abdomen management, and recurrent gastrojejunal leakage. After failure of repeated surgical interventions, EndoVAC therapy was initiated as salvage treatment. A manually assembled system using a white open-pore polyurethane foam sponge was endoscopically placed and exchanged over 22 days, achieving complete clinical and endoscopic resolution without further surgical intervention. This case highlights the role of EndoVAC as an effective salvage strategy in complex postoperative leaks following revisional bariatric surgery.

## Introduction

Anastomotic leaks remain one of the most severe complications in gastrointestinal and bariatric surgery, associated with significant morbidity and mortality. Management is particularly challenging in revisional surgery due to altered anatomy, fibrosis, and impaired tissue perfusion.

Endoluminal vacuum therapy (EVT) is a minimally invasive technique that applies continuous negative pressure to promote drainage, reduce contamination, collapse cavities, and stimulate granulation tissue formation [1, 2]. Systematic reviews and meta-analyses report pooled clinical success rates of ~80%–85% [3, 4].

Comparative studies suggest that EVT may achieve higher treatment success and lower complication rates than self-expanding metal stents, which have reported success rates ranging from 60% to 70% in complex leaks [5].

However, evidence regarding EVT in the setting of revisional bariatric surgery and repeated surgical failure remains limited.

## Case report

A 55-year-old woman with a history of sleeve gastrectomy performed 16 years earlier underwent one-anastomosis gastric bypass due to obstructive symptoms.

The postoperative course was complicated by dehiscence of the gastrojejunal anastomosis, requiring multiple reinterventions, open abdomen management, placement of a feeding jejunostomy, and prolonged intensive care unit admission.

**Figure 1:**
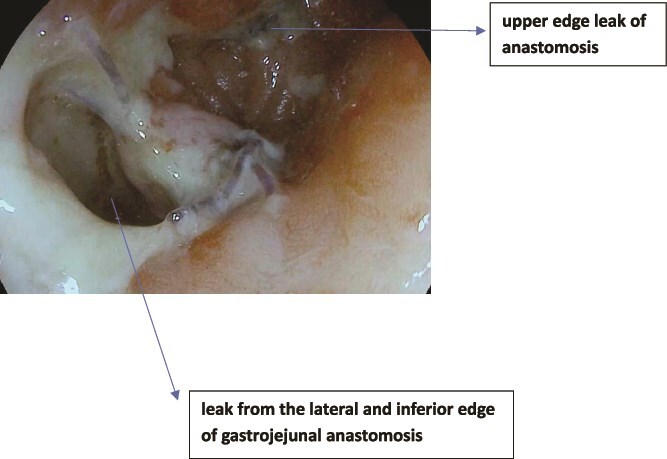
Endoscopic image showing a gastrojejunal anastomotic defect with an open leak cavity before initiation of EVT.

Three months later, the patient developed multiple gastrocutaneous fistulas, necessitating complex open reconstruction with a Roux-en-Y bypass. Despite this intervention, a recurrent anastomotic leak was diagnosed on postoperative day 7 (Fig. 1).

**Figure 2:**
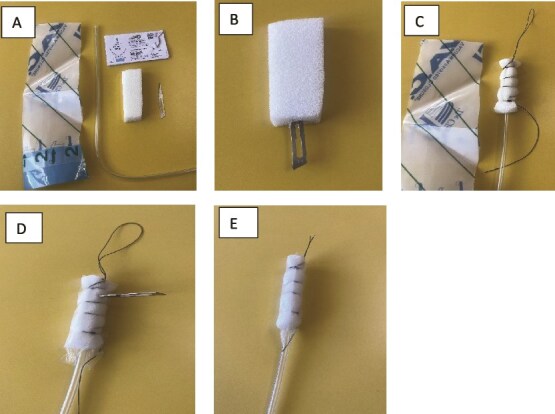
Assembly of EVT (A) materials: No. 11 scalpel, 16 Fr nasogastric tube, 1–0 silk suture, 8 × 8 cm Steri-drape, and 2 × 4 cm white foam. (B) Cut the foam in half. (C) Insert the nasogastric tube into the foam and secure it with 1–0 silk suture. Cover with Steri-drape. (D) Create small perforations along the tube within the foam to complete the homemade EVT (E).

Given repeated surgical failure and high operative risk, endoluminal vacuum therapy was initiated as salvage treatment (Fig. 2).

Following the diagnosis of recurrent gastrojejunal anastomotic leakage on postoperative day (POD) 7, EVT was initiated during the same endoscopic procedure. The device was positioned within the leak cavity under direct endoscopic visualization, and continuous negative pressure of −125 mmHg was applied.

The first device exchange was performed on POD 11, corresponding to 4 days after the initial EVT placement, because minimal output was observed in the vacuum canister, raising concern regarding reduced effectiveness of the system. Endoscopic evaluation demonstrated a marked decrease in the size of the cavity, healthy granulation tissue, and no evidence of necrotic debris or purulent collections. The device was exchanged and repositioned.

The second EVT exchange was performed 7 days after the initial placement. Endoscopy revealed continued contraction of the cavity with progressive granulation tissue formation and further reduction of the anastomotic defect. Given the favorable evolution, a new EVT device was inserted to continue therapy.

A third device exchange was performed on day 14 after the initial placement, demonstrating near-complete collapse of the cavity with healthy granulation tissue and minimal residual defect. Continuous negative-pressure therapy was maintained.

**Figure 3:**
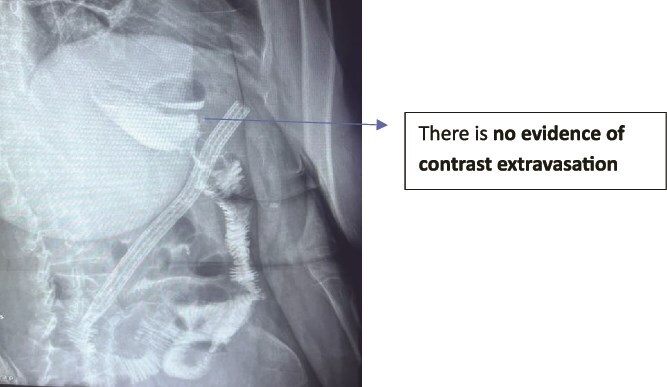
Gastrointestinal transit showing no evidence of contrast leak.

**Figure 4:**
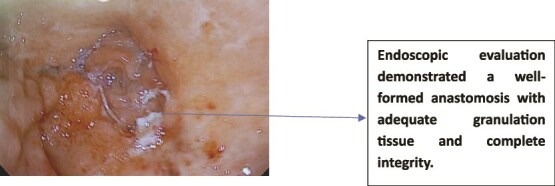
Complete closure of the defect after 22 days of therapy.

On Day 20 after the initial placement, an upper gastrointestinal contrast study demonstrated complete resolution of the leak without evidence of contrast extravasation (Fig. 3). A final endoscopic evaluation was performed on Day 22, confirming complete epithelialization and closure of the anastomotic defect (Fig. 4). The EVT system was removed during the same procedure.

Following confirmation of leak closure, oral intake was gradually resumed with a liquid diet, which was well tolerated, allowing discontinuation of total parenteral nutrition. The patient was subsequently discharged in good clinical condition and remains asymptomatic during outpatient follow-up, with satisfactory oral intake and no evidence of recurrent leakage (Table 1).

**Table 1:** Timeline of clinical course and endoluminal vacuum therapy

Time	Clinical findings	Intervention
POD 7	Recurrent gastrojejunal anastomotic leak confirmed by upper endoscopy.	Initial EVT placement, nil per os (NPO), 100% total parenteral nutrition (TPN), broad-spectrum intravenous antibiotics, continuous negative-pressure therapy (−125 mmHg).
Day 4 after EVT placement (POD 11)	Minimal output in the vacuum canister. Endoscopy showed reduction of the cavity with healthy granulation tissue and no necrotic debris.	First EVT exchange; continuation of 100% TPN and broad-spectrum intravenous antibiotics.
Day 7 after EVT placement	Progressive contraction of the cavity with healthy granulation tissue.	Second EVT exchange; continued EndoVAC therapy, TPN, and antibiotic treatment.
Day 14 after EVT placement	Near-complete collapse of the cavity with minimal residual defect.	Third EVT exchange; completion of broad-spectrum antibiotic therapy.
Day 20 after EVT placement	Upper gastrointestinal contrast study showed no evidence of contrast extravasation.	TPN reduced to 50% of nutritional requirements while maintaining EVT therapy.
Day 22 after EVT placement	Follow-up endoscopy confirmed complete closure and epithelialization of the defect.	EVT removal; initiation of a liquid diet; progressive discontinuation of TPN.
Follow-up	Good clinical recovery with adequate oral intake and no recurrence of leakage.	Hospital discharge and outpatient follow-up.

## Endoluminal vacuum therapy technique

The EVT device was manually assembled using a 16-Fr silicone nasogastric tube and a 3-cm white open-pore polyurethane foam sponge. The sponge was secured to the distal end of the tube with 1–0 silk sutures and covered with a sterile fenestrated adhesive drape to ensure uniform distribution of negative pressure. Additional fixation sutures were placed at both the proximal and distal ends to facilitate endoscopic manipulation and accurate positioning within the leak cavity (Fig. 2).

The device was introduced endoscopically under direct visualization and positioned within the leak cavity. Continuous negative pressure of −125 mmHg was applied using a standard vacuum therapy system. At each scheduled device exchange, the cavity was irrigated and endoscopically reassessed before placement of a new EVT system until complete closure of the defect was achieved.

## Discussion

Endoscopic vacuum therapy has emerged as a highly effective modality for the treatment of gastrointestinal anastomotic leaks. Meta-analyses report pooled clinical success rates of ~80%–85%, with favorable safety profiles [3, 4].

When compared with alternative endoscopic approaches, particularly self-expanding metal stents (SEMS), EVT appears to provide superior outcomes in complex leaks [6, 7]. Reported success rates for SEMS range between 60% and 70%, with limitations including stent migration, inadequate drainage of infected cavities, and persistent sepsis [5]. In contrast, EVT enables continuous drainage, promotes cavity collapse, and enhances granulation tissue formation, addressing key pathophysiological mechanisms of leak persistence.

Comparative meta-analyses have demonstrated significantly higher treatment success with EVT, with an odds ratio of ~2.5 in favour of EVT over stenting, along with lower complication rates and reduced need for reintervention [5].

In addition to endoscopic approaches, surgical reintervention in this setting is associated with high morbidity and often limited success, particularly in revisional bariatric surgery where tissue quality is compromised.

In the present case, EVT was successfully used after multiple surgical failures in a highly complex revisional bariatric patient with prior open abdomen management and fistula formation. This clinical scenario represents a particularly high-risk subset in which conventional strategies frequently fail.

In this case, a white open-pore polyurethane foam sponge was used, which may be advantageous in smaller cavities or in proximity to fragile tissue due to its lower adherence compared with black foam, potentially reducing mucosal trauma during device exchanges.

This case supports the role of EVT as a definitive salvage therapy, capable of achieving leak closure while avoiding further high-risk surgical interventions.
